# Clinical Characteristics and Results of Semen Tests Among Men With Coronavirus Disease 2019

**DOI:** 10.1001/jamanetworkopen.2020.8292

**Published:** 2020-05-07

**Authors:** Diangeng Li, Meiling Jin, Pengtao Bao, Weiguo Zhao, Shixi Zhang

**Affiliations:** 1 Nanlou Respiratory Diseases Department, Chinese People’s Liberation Army General Hospital, Beijing, China; 2 Department of Nephrology, Chinese People’s Liberation Army General Hospital, Chinese People’s Liberation Army Institute of Nephrology, State Key Laboratory of Kidney Diseases, National Clinical Research Center for Kidney Diseases, Chinese People’s Liberation Army Postgraduate Medical School, Beijing, China; 3 Department of Nephrology, Beijing-Chaoyang Hospital, Beijing, China; 4 Department of Respiratory Medicine, The Eighth Medical Center of Chinese People’s Liberation Army General Hospital, Beijing, China; 5 Shangqiu Municipal Hospital, Shangqiu, China

## Abstract

This cohort study examines the clinical characteristics of men with coronavirus disease 2019 whose semen tested positive for severe acute respiratory syndrome coronavirus 2.

## Introduction

In December 2019, an outbreak of pneumonia associated with coronavirus disease 2019 (COVID-19) occurred in Wuhan, China, and rapidly spread to other parts of China and overseas.^1^ It has been confirmed that COVID-19 has the characteristic of human-to-human transmission, mainly through respiratory droplets and contact. Other routes require further verification. The virus responsible for COVID-19, severe acute respiratory syndrome coronavirus 2 (SARS-CoV-2) has been detected in stool, gastrointestinal tract, saliva, and urine samples.^2^ However, little is known about SARS-CoV-2 in semen.

## Methods

This cohort study was performed after patients gave written informed consent for research purposes, and in compliance with the Helsinki Declaration^3^ with the approval of the ethics committee of Shangqiu Municipal Hospital, Shangqiu, China. This study is reported following the Strengthening the Reporting of Observational Studies in Epidemiology (STROBE) reporting guideline.

We identified all male patients with laboratory-confirmed COVID-19 aged 15 years and older between January 26, 2020, and February 16, 2020, in Shangqiu Municipal Hospital, which is the only designated hospital for the treatment of COVID-19 in Shangqiu, in the east of Henan province. Following guidance from the World Health Organization,^4^ laboratory confirmation for COVID-19 was defined as positive result for SARS-CoV-2 in real-time reverse transcriptase–polymerase chain reaction assay of nasal and pharyngeal swabs.^1^ Enrolled patients were asked to provide a semen sample for SARS-CoV-2 testing.

Groups were compared using the *t* test, χ^2^ test, or Mann-Whitney or Kruskal-Wallis test. All statistical analyses were performed using SPSS statistical software version 19 (IBM). *P* values were 2-tailed, and *P* < .05 was considered to indicate significant differences.

## Results

Among 50 patients identified, 12 patients were unable to provide a semen specimen because of erectile dysfunction, being in a comatose state, or dying prior to recruitment; therefore, a total of 38 patients were enrolled for semen testing. Of these 38 participants who provided a semen specimen, 23 participants (60.5%) had achieved clinical recovery and 15 participants (39.5%) were at the acute stage of infection. Results of semen testing found that 6 patients (15.8%) had results positive for SARS-CoV-2, including 4 of 15 patients (26.7%) who were at the acute stage of infection and 2 of 23 patients (8.7%) who were recovering, which is particularly noteworthy. But there was no significant difference between negative and positive test results for patients by age, urogenital disease history, days since onset, days since hospitalization, or days since clinical recovery. The clinical characteristics of patients with positive test results for SARS-CoV-2 in semen are shown in the Table.

**Table.** Clinical Characteristics of Patients With Positive Test Results for Severe Acute Respiratory Syndrome Coronavirus 2 in Semen

Patient^a^	Approximate age, y^a^	Time since onset of symptoms, d	Time since hospitalization, d	Time since clinical recovery, d	Presence of urogenital disease	Other comorbidity
1	20s	6	2	NA^b^	No	Coronary heart disease, hypertension
2	20s	10	6	NA^b^	No	Coronary heart disease
3	30s	11	5	NA^b^	No	No
4	40s	9	8	NA^b^	No	No
5	50s	12	10	2	Yes	No
6	30s	16	13	3	No	Chronic bronchitis

Abbreviation: NA, not applicable.

^a^ For the purpose of anonymity, patients are identified by number and their ages are given as approximates.

^b^ Patient was still in the acute stage of infection.

## Discussion

In this cohort study, we found that SARS-CoV-2 can be present in the semen of patients with COVID-19, and SARS-CoV-2 may still be detected in the semen of recovering patients. Owing to the imperfect blood-testes/deferens/epididymis barriers, SARS-CoV-2 might be seeded to the male reproductive tract, especially in the presence of systemic local inflammation. Even if the virus cannot replicate in the male reproductive system, it may persist, possibly resulting from the privileged immunity of testes. So far, researchers have found 27 viruses associated with viremia in human semen. But the presence of viruses in semen may be more common than currently understood, and traditional non–sexually transmitted viruses should not be assumed to be totally absent in genital secretions.^5,6^ Studies on viral detection and semen persistence are beneficial to clinical practice and public health, especially concerning viruses that could cause high mortality or morbidity, such as SARS-CoV-2.

This study is limited by the small sample size and the short subsequent follow-up. Therefore, further studies are required with respect to the detailed information about virus shedding, survival time, and concentration in semen.

If it could be proved that SARS-CoV-2 can be transmitted sexually in future studies, sexual transmission might be a critical part of the prevention of transmission, especially considering the fact that SARS-CoV-2 was detected in the semen of recovering patients. Abstinence or condom use might be considered as preventive means for these patients. In addition, it is worth noting that there is a need for studies monitoring fetal development. Therefore, to avoid contact with the patient’s saliva and blood may not be enough, since the survival of SARS-CoV-2 in a recovering patient’s semen maintains the likelihood to infect others. Our study might contribute by providing new information to the current discourse regarding COVID-19 prevention and control.
