# Error in Methods

**DOI:** 10.1001/jamanetworkopen.2020.10845

**Published:** 2020-06-01

In the original investigation titled “Clinical Characteristics and Results of Semen Tests Among Men With Coronavirus Disease 2019,”^1^ published May 7, 2020, the Methods section did not specify the method used to detect severe acute respiratory syndrome coronavirus 2 in semen. The method was reverse transcriptase–polymerase chain reaction assay. This article has been corrected.

## References

[1] Li D, Jin M, Bao P, Zhao W, Zhang S Clinical characteristics and results of semen tests among men with coronavirus disease 2019. JAMA Netw Open. 2020;3(5):e208292. doi:10.1001/jamanetworkopen.2020.8292 32379329 PMC7206502

